# Eribulin, Child-Pugh score, and liver-function tests: lessons from pivotal breast cancer studies 301 and 305

**DOI:** 10.1186/s13058-021-01407-w

**Published:** 2021-03-18

**Authors:** Iain R. Macpherson, Yaohua He, Carlo Palmieri

**Affiliations:** 1 Institute of Cancer Sciences, CR-UK Beatson Institute, University of Glasgow, Glasgow, UK; 2 Formerly of Eisai Inc., Woodcliff Lake, NJ USA; 3 Department of Molecular and Clinical Cancer Medicine, The Institute of Systems, Molecular and Integrative Biology, University of Liverpool, Ashton Street, Liverpool, L69 3GE UK; 4 Academic Department of Medical Oncology, The Clatterbridge Cancer Centre NHS Foundation Trust, Liverpool, UK

**Keywords:** Breast cancer, Eribulin, Bilirubin, Liver-function tests, Child-Pugh score

## Abstract

**Background:**

The recommended starting dose of eribulin in patients with hepatic impairment is based on the Child-Pugh score, largely informed by a pharmacokinetic study of 18 patients. In the pivotal studies of eribulin in metastatic breast cancer (Study 301 and Study 305 [EMBRACE]), entry criteria and dose modifications were based on liver-function test (LFT) results rather than Child-Pugh score. In populations such as patients with metastatic breast cancer, in which metastatic infiltration is the predominant cause of hepatic impairment, using Child-Pugh score may be problematic; in clinical practice, it has been more common for oncologists to make dosing decisions based on LFTs. To address this, the effects of abnormal baseline LFT results on eribulin efficacy and safety were investigated.

**Methods:**

In this pooled post hoc analysis, 1062 patients who were randomized to receive eribulin in Studies 301 and 305 were divided into 4 groups: (A) no elevated LFT results (no liver impairment); (B) increased levels of aspartate aminotransferase and/or alanine aminotransferase; (C) decreased albumin and/or increased levels of aspartate aminotransferase and/or alanine aminotransferase but not increased bilirubin; and (D) increased bilirubin. Patients were subcategorized by presence of liver metastasis. Drug exposure, dose intensity, and treatment-emergent adverse events (TEAEs) were analyzed.

**Results:**

Eribulin mesylate mean dosage was 0.82 (group A)–0.65 mg/m^2^/week (group D). Group D had shorter treatment, more dose reductions/delays, more TEAEs leading to dose modifications, and numerically lower objective response rates and clinical benefit rates versus groups A–C. TEAE rates leading to dose modification were similar between group D (45.5%) and groups A–C (range, 43.5–54.9%) in the absence of liver metastases, but higher in group D (91.3%) compared with groups A–C (range, 41.7–54.3%) if liver metastases were present.

**Conclusions:**

Mild elevations in bilirubin levels were associated with increased toxicity and a greater requirement for dose modifications. Based both on these study data and existing recommendations, we propose a novel scheme to guide initial dose selection in patients with metastatic breast cancer and hepatic impairment that is based on LFTs rather than Child-Pugh score.

**Supplementary Information:**

The online version contains supplementary material available at 10.1186/s13058-021-01407-w.

## Introduction

Eribulin, an inhibitor of microtubule dynamics [1–3], has demonstrated antitumor activity in metastatic breast cancer (MBC) in two randomized phase 3 trials. In previously treated (2–5 chemotherapeutic regimens in EMBRACE/Study 305 [NCT00388726] and ≤ 3 chemotherapeutic regimens in Study 301 [NCT00337103]) women with MBC, EMBRACE demonstrated that eribulin significantly improved overall survival (OS) versus physician’s choice of treatment [4], whereas Study 301 did not demonstrate that eribulin was superior to capecitabine with regard to OS [5]. As a result of these studies (Study 305 in the USA; Studies 305 and 301 in the European Union), eribulin is approved for the treatment of patients with MBC who previously received ≥ 1 (European Union) or ≥ 2 (USA) prior chemotherapies for the treatment of MBC, including an anthracycline and a taxane in the adjuvant or metastatic setting [6, 7].

The main route of elimination of eribulin is via biliary excretion [8]. Although eribulin seems to be metabolized by CYP3A4 in vitro [9], it is excreted predominately as unchanged drug in patients [8]. Because eribulin features hepatic excretion and metastatic disease commonly involves the liver, the pharmacokinetics (PK) of eribulin in 18 patients with solid tumors and hepatic impairment were assessed [10]. In this study, patients were divided into 1 of 3 groups: normal liver function, abnormal liver function and Child-Pugh class A, or abnormal liver function and Child-Pugh class B. The eribulin dose was based on the patient’s Child-Pugh class. Patients who had normal hepatic function received eribulin mesylate 1.4 mg/m^2^ (equivalent to eribulin 1.23 mg/m^2^ [expressed as free base]) whereas patients who were classified as Child-Pugh class A or B received a reduced starting dose of 1.1 mg/m^2^ and 0.7 mg/m^2^, respectively. Compared with patients who had normal liver function, mean dose-normalized area under the curve from zero to infinity (AUC_0-∞_) was 1.75-fold (90% CI 1.15–2.66) higher in patients classified as Child-Pugh class A and 2.48-fold (90% CI 1.57–3.92) higher in patients classified as Child-Pugh class B [10]. Based on this study, a reduced eribulin dosage is recommended for Child-Pugh class A or B hepatic impairment (1.1 mg/m^2^ or 0.7 mg/m^2^ of eribulin mesylate, respectively, equivalent to eribulin 0.97 mg/m^2^ and 0.62 mg/m^2^ [expressed as free base]) [6, 7].

The Child-Pugh score—derived from clinical and biochemical features—was developed to assess the prognosis in patients with chronic liver disease [11, 12]. The score was not designed, and is not validated, for deciding the dose of systemic cancer therapies in patients with liver dysfunction in the context of metastatic cancer. The European Medicines Agency (EMA) and US Food and Drug Administration (FDA) have noted the lack of biomarkers available for categorizing liver impairment with respect to effects on drug PK and have therefore created guidance on the use of Child-Pugh scores [13, 14]. An important caveat in this guidance is that the abnormalities in factors comprising the Child-Pugh score should be directly attributable to liver dysfunction and not to an alternative cause; however, in the context of MBC, this distinction may not be readily evident. As such, the challenges of dose selection for anticancer drugs in the setting of hepatic impairment are widely recognized [15].

In clinical practice, dosing decisions for chemotherapeutic agents that are metabolized or cleared via the liver are generally based on baseline liver-function test (LFT) results rather than Child-Pugh score. Of note, inclusion criteria regarding hepatic function within Studies 301 and 305 were defined by levels of total bilirubin and transaminases and not Child-Pugh score. However, no published data are available regarding the relationship between LFT findings and the efficacy and safety of eribulin. As such, this analysis of pooled efficacy and safety data based on baseline LFT results was undertaken.

## Materials and methods

### Studies

This retrospective, post hoc, pooled analysis used data from 2 prospective studies of eribulin in MBC [4, 5]. In Study 305/EMBRACE, women with locally recurrent disease or MBC (2–5 previous chemotherapy regimens, including an anthracycline and a taxane) were randomly assigned 2:1 to eribulin mesylate 1.4 mg/m^2^ intravenously (equivalent to 1.23 mg/m^2^ eribulin [expressed as free base]) on days 1 and 8 every 21 days or a treatment of the physician’s choice [4]. To be eligible, patients were required to have adequate liver function in screening blood tests as evidenced by bilirubin levels ≤ 1.5 times the upper limits of normal (ULN) and alkaline phosphatase (AP), alanine aminotransferase (ALT), and aspartate aminotransferase (AST) levels ≤ 3 × ULN (in the case of liver metastases ≤ 5 × ULN). If bone metastases were present, liver-specific AP was separated from the total and used to assess liver-function instead of total AP.

In Study 301, patients with MBC (≤ 3 prior chemotherapy regimens, of which ≤ 2 were for advanced or metastatic disease, and prior therapy with an anthracycline and a taxane) were randomly assigned 1:1 to eribulin mesylate 1.4 mg/m^2^ intravenously (equivalent to 1.23 mg/m^2^ eribulin [expressed as free base]) on days 1 and 8 of each 21-day cycle versus capecitabine 1.25 g/m^2^ twice daily on days 1–14 of each 21-day cycle as first-, second-, or third-line chemotherapy for advanced breast cancer [5]. Adequate liver function was defined as per Study 305, except in the case of bone metastases, in which liver-specific AP was ≤ 3 × ULN. Both studies required patients to have an Eastern Cooperative Oncology Group performance status of 0–2.

Both Study 301 and Study 305 were conducted in accordance with local laws, the Declaration of Helsinki, and the International Conference on Harmonization Good Clinical Practice guidelines, and with the approval of each Institutional Review Board. All patients provided written informed consent.

### Pooled analysis

Pooled data from eribulin-treated patients in Study 301 and Study 305 [16] were analyzed by liver impairment subgroups. Liver impairment was defined using the Common Terminology Criteria for Adverse Events (CTCAE) as an increased baseline level (≥ grade 1) of bilirubin, ALT, or AST levels or a decreased baseline level of albumin. Patients were then allocated to 1 of 4 subgroups: group A (no liver impairment), group B (increased AST and/or ALT levels only), group C (decreased albumin and/or increased AST, and/or ALT levels but not increased bilirubin), and group D (increased bilirubin). Liver involvement was defined as presence of target or non-target liver lesions at baseline.

Safety analyses included drug exposure, dose intensity, and dose modification (i.e., dose interruptions, reductions, and delays). Treatment-emergent adverse events (TEAEs) by CTCAE grade and TEAEs leading to dose modification were assessed. TEAE terms were coded using Medical Dictionary for Drug Regulatory Affairs, version 14.1, and graded using CTCAE, version 3.0. Absolute neutrophil count, change from baseline, and neutrophil/granulocyte CTCAE grade by visit were also evaluated. The safety population comprised patients who received any dose of study drug and was used to summarize the safety data.

OS data were summarized using the intent-to-treat (ITT) population (all randomized patients). Median OS in months and 1-, 2-, and 3-year survival rates (and 95% CIs) were calculated using Kaplan-Meier estimate and Greenwood’s formula. Tumor assessment data were summarized using the ITT population from Study 301, plus the evaluable population from Study 305 (all patients with measurable disease). This population was used to calculate the point estimates and 2-sided 95% CIs using the exact Clopper-Pearson method for objective response rate (ORR; defined as complete response [CR] + partial response [PR]) and clinical benefit rate (CBR; defined as CR + PR + stable disease ≥ 6 months). Summaries of ORR and CBR were based on tumor response data from independent review.

## Results

### Clinicopathologic and liver impairment data

Overall patient demographics and baseline disease characteristics for the pooled analysis have been published elsewhere [16]. In the safety population, this post hoc analysis included patients at baseline with normal LFTs (AST/ALT, albumin and bilirubin) (group A; *n =* 540), increased AST and/or ALT levels only (group B; *n =* 292), increased AST and/or ALT and/or decreased albumin levels (group C; *n =* 440), and increased bilirubin (group D; *n =* 34). The following values are the number of patients with and without metastatic liver involvement, respectively, by group: group A (*n =* 204 and *n =* 336), group B (*n =* 210 and *n =* 82), group C (*n =* 294 and *n =* 146), and group D (*n =* 23 and *n =* 11).

In the ITT population, additional patients were included in groups A–D: patients with no liver impairment (group A, *n* = 546), increased AST and/or ALT levels only (group B, *n =* 294), any abnormality except increased bilirubin (group C, *n =* 443), and increased bilirubin (group D, *n =* 34).

### Exposure

The planned eribulin mesylate dosage intensity was 0.933 mg/m^2^/week. The mean actual dosage intensity was 0.82 mg/m^2^/week for group A, 0.78 mg/m^2^/week for both groups B and C, and 0.65 mg/m^2^/week for group D (Table 1). The dosage intensity remained similar for each liver impairment group when analyzed by metastatic liver involvement (Supplementary Table S1). Specifically, in patients with no liver involvement, dosage intensity ranged from 0.81 mg/m^2^/week for group A to 0.67 mg/m^2^/week for group D and in patients with liver involvement, dosage intensity was 0.83 mg/m^2^/week for group A to 0.64 mg/m^2^/week for group D.

**Table 1:** Eribulin mesylate exposure and dose modifications in patients with liver impairment (safety population)

Parameter	Normal	Liver impairment		
	Group A (***n*** = 540)	Group B (***n*** = 292)	Group C (***n*** = 440)	Group D (***n*** = 34^**a**^)
Bilirubin, μmol/L, median (range)	8.55 (1.6, 19.9)	8.55 (1.7, 21.0)	8.55 (1.7, 23.9)	22.70 (16.0, 75.2)
AST, U/L, median (range)	23.0 (2.6, 48.0)	51.0 (9.6, 323.0)	47.0 (9.2, 369.0)	55.0 (16.0, 474.0)
ALT, U/L, median (range)	19.0 (1.3, 51.0)	45.5 (5.0, 407.0)	39.0 (5.0, 407.0)	36.0 (12.0, 220.0)
Albumin, g/L, median (range)	42.0 (29.0, 66.0)	41.2 (31.0, 58.9)	39.0 (18.0, 58.9)	42.0 (28.0, 63.3)
Mean duration of treatment, days (SD)	166.12 (163.44)	151.65 (122.50)	140.26 (113.30)	128.76 (75.90)
Median number of cycles completed on study (range)	6.0 (1.0, 65.0)	6.0 (1.0, 38.0)	5.0 (1.0, 38.0)	4.5 (1.0, 15.0)
Mean actual dose intensity of eribulin mesylate, mg/m^2^/week (SD)	0.82 (0.14)	0.78 (0.16)	0.78 (0.16)	0.65 (0.18)
Patients with a dose delay, *n* (%)	223 (41.3)	141 (48.3)	212 (48.2)	23 (67.7)
Patients with a dose reduction, *n* (%)	141 (26.1)	101 (34.6)	153 (34.8)	21 (61.8)

^a^*n* = 33 for mean actual dose intensity analyses

Group A, no liver impairment; group B, increased AST and/or ALT only; group C, any abnormality except increased bilirubin; group D, increased bilirubin

*ALT*, alanine aminotransferase; *AST*, aspartate aminotransferase; *SD*, standard deviation

Compared with patients in group A, patients in group D received treatment for a shorter time (mean duration of treatment: 166 vs 129 days, respectively), and a larger percentage of patients experienced dose reductions (26.1% vs 61.8%, respectively) or dose delays (41.3% vs 67.7%, respectively) (Table 1). These same trends were also observed when eribulin exposure was analyzed by whether liver involvement was present. In patients with no liver involvement, dose delays were experienced by 41.7% of patients in group A and 54.6% of patients in group D, and dose reductions were experienced by 27.7% and 54.6% of patients in groups A and D, respectively (Supplementary Table S1). For patients with liver involvement, dose delays and reductions, respectively, were experienced by 40.7% and 23.5% of patients in group A and by 73.9% and 65.2% of patients in group D (Supplementary Table S1).

### Safety

TEAEs were experienced by ≥ 95% of patients in all groups (Supplementary Table S2). The most commonly reported TEAEs (occurring in ≥ 30% of patients in any group) were neutropenia, alopecia, nausea, and leucopenia (Supplementary Table S2). Among patients in groups A, B, C, and D, total TEAEs leading to dose modifications occurred in 42.8%, 54.5%, 52.5%, and 76.5% of patients respectively; grade 3 or 4 TEAEs leading to dose modifications were reported in 32.6%, 42.1%, 41.8%, and 70.6% of patients, respectively (Table 2). Patients in group D had a higher incidence of grade ≥ 3 TEAEs leading to dose modification than patients in group A, B, or C. The most common TEAEs leading to dose reduction or delay were neutropenia and leukopenia, which were more common among patients in group D (58.8% and 17.7%, respectively) compared with all other groups (Table 2). Among patients in groups A, B, C, and D, febrile neutropenia leading to dose modifications occurred in 2%, 2%, 3%, and 6% of patients, respectively. Although the sample size in group D was small (*n =* 34), liver involvement appeared to change the pattern of TEAEs that led to dose modifications. The number of patients with TEAEs leading to dose modifications was similar across groups within the subset of patients without liver involvement (group A: 43.5%; group B: 54.9%; group C: 52.7%; group D: 45.5%). However, for patients with liver involvement, the number of TEAEs leading to dose modifications were markedly higher in those with elevated bilirubin compared with other groups (group A: 41.7%; group B: 54.3%; group C: 52.4%; group D: 91.3%; Supplementary Table S3).

**Table 2:** Number of patients experiencing TEAEs leading to dose modifications (reductions, delays, or interruptions) by CTCAE grade, and occurring in > 10% of patients in any group (safety population)

	Normal	Liver impairment		
***n*** (%)	Group A (***n*** = 540)	Group B (***n*** = 292)	Group C (***n*** = 440)	Group D (***n*** = 34)
**Total**	231 (42.8)	159 (54.5)	231 (52.5)	26 (76.5)
**Grade 1**	8 (1.5)	11 (3.8)	13 (3.0)	2 (5.9)
**Grade 2**	47 (8.7)	25 (8.6)	34 (7.7)	0
**Grade 3**	111 (20.6)	76 (26.0)	114 (25.9)	10 (29.4)
**Grade 4**	65 (12.0)	47 (16.1)	70 (15.9)	14 (41.2)
**Neutropenia**	125 (23.2)	91 (31.2)	132 (30.0)	20 (58.8)
**Leucopenia**	44 (8.2)	26 (8.9)	39 (8.9)	6 (17.7)

Group A, no liver impairment; group B, increased AST and/or ALT only; group C, any abnormality except increased bilirubin; group D, increased bilirubin

*ALT*, alanine aminotransferase; *AST*, aspartate aminotransferase; *CTCAE*, Common Terminology Criteria for Adverse Events; *TEAE*, treatment-emergent adverse event

In all groups, the number of patients experiencing neutrophil/granulocyte counts of grade ≥ 1 TEAEs was 29% to 39% on day 8 of cycle 1, and reduced to 14 to 22% on day 8 of subsequent treatment cycles (Supplementary Table S4).

### Efficacy

ORR was 11.6% in group A, 14.0% in group B, and 12.7% in group C (Table 3). CBR was 27.2% in group A, 24.6% in group B, and 22.2% in group C (Table 3). However, the group with an increased bilirubin at baseline (group D) was associated with numerically lower ORR (0%) and CBR (19%) compared with all other groups (Table 3). The median OS was numerically lower in patients with liver impairment (13.2, 12.3, and 12.3 months in groups B, C, and D, respectively) compared with patients without liver impairment (group A: 17.5 months; Supplementary Table S5). OS in patient subgroups by liver involvement is summarized in Supplementary Table S6.

**Table 3:** Response rates in patients with liver impairment (independent review)

	Normal	Liver impairment		
***n*** (%)	Group A (***n =*** 533)	Group B (***n*** = 285)	Group C (***n =*** 424)	Group D (***n*** = 32)
**CR**	3 (0.6)	1 (0.4)	1 (0.2)	0
**PR**	59 (11.1)	39 (13.7)	53 (12.5)	0
**SD**	275 (51.6)	141 (49.5)	199 (46.9)	24 (75.0)
**Progressive disease**	165 (31.0)	82 (28.8)	137 (32.3)	6 (18.8)
**Not evaluable**	6 (1.1)	10 (3.5)	16 (3.8)	1 (3.1)
**Unknown**^**a**^	25 (4.7)	12 (4.2)	18 (4.2)	1 (3.1)
**Objective response rate (CR + PR)**	62 (11.6)	40 (14.0)	54 (12.7)	0
**95% CI**^**b**^	(9.0, 14.7)	(10.2, 18.6)	(9.7, 16.3)	(0, 10.9)
**Clinical benefit rate (CR + PR + SD**^**c**^**)**	145 (27.2)	70 (24.6)	94 (22.2)	6 (18.8)
**95% CI**^**b**^	(23.5, 31.2)	(19.7, 30.0)	(18.3, 26.4)	(7.2, 36.4)

^a^Patients who had no baseline scans or only baseline scans

^b^Exact Clopper-Pearson 2-sided CI

^c^For clinical benefit, SD must be at least 6 months

Group A, no liver impairment; group B, increased AST and/or ALT only; group C, any abnormality except increased bilirubin; group D, increased bilirubin

*ALT*, alanine aminotransferase; *AST*, aspartate aminotransferase; *CI*, confidence interval; *CR*, complete response; *PR*, partial response; *SD*, stable disease

## Discussion

Results from this retrospective, post hoc analysis of pooled data on eribulin suggest that even mild elevations in bilirubin at baseline were associated with a reduction in dose intensity, more frequent early-dose reduction, and a higher proportion of grades 3 and 4 toxicity, compared with patients with normal LFT results and those solely with increases in levels of transaminases and/or decreased albumin. This was evident despite study-entry criteria limiting enrolment to patients with bilirubin no greater than 1.5 times upper limit of normal. Although these results are based on a relatively small number of patients, the data are consistent with, and can be explained by, the published PK and pharmacology data for eribulin. In vivo studies have shown that eribulin’s CYP3A4 metabolism is of limited contribution, as evidenced by the observation that coadministration of eribulin with a CYP3A4 inhibitor or inducer has no effect on its exposure or systemic clearance [17–19], as well as the predominant excretion of eribulin as unchanged drug. Pooled data from 513 eribulin-treated patients have shown that eribulin clearance decreased with increasing AP and bilirubin [20], which is consistent with eribulin’s known biliary route of excretion [8]. Taken together, disruption or obstruction to the biliary tree, as indicated by an elevated bilirubin, is likely to lead to impaired excretion of eribulin with increased AUC and greater toxicity with subsequent impact on the delivery of dosage intensity. In this analysis, we focused on bilirubin alone because the etiology of raised AP may be difficult to determine in a population with a high incidence of skeletal metastases. The current guidance regarding eribulin dosing in MBC is based on Child-Pugh score and derived from a relatively small PK study of 18 patients with advanced cancer [10]. Within this study, the median bilirubin level in the Child-Pugh class A group was similar to the normal liver-function group; however, 42% of patients in this group had an additional hepatic pathology, and all had liver metastasis. The impact of these hepatic pathologies on the PK findings within the Child-Pugh class A group is not clear and cannot be discounted or ascertained from the data presented. Higher levels of baseline bilirubin were present within the Child-Pugh class B group, and this may explain some of the observed PK differences between group A and group B [10]. Of note, Child-Pugh criteria were not used in any of the phase 3 eribulin studies as part of the assessments for study entry—these studies used baseline LFT parameters [4, 5]. Given these data, we have explored dosing decisions based on bilirubin level and the presence of liver metastasis rather than Child-Pugh criteria.

Of specific concern, the Child-Pugh score was developed to evaluate operative risk in patients with chronic liver disease; however, the Child-Pugh score is now commonly used as a prognostic tool in these patients [12]. Despite having several limitations, the Child-Pugh score is described in EMA and FDA guidance as a methodology for assessing hepatic function for the evaluation of the PK of medicinal products [13, 14]. The EMA recognizes that the Child-Pugh criteria were not developed for the purpose of predicting drug-elimination capacity, and if this criteria is used, researchers must ensure that patients included in the study have an adequate range of decrease in serum albumin and increase in serum bilirubin and prothrombin time [13].

In patients with solid tumors, abnormal liver function is often caused by metastatic disease. For example, whereas the incidence of cirrhotic liver disease in the population with breast cancer is relatively low (e.g., 0.3% in American women with breast cancer aged ≥ 66 years) [21], metastatic spread to the liver is common, with liver involvement present in approximately 25% of women presenting with metastatic disease [22]. Within this population, the use of Child-Pugh score is problematic and is likely to lead to inconsistencies in dose selection with the potential for over- or under-treatment. As clear guidance is lacking as to when the Child-Pugh score should be calculated in routine oncological practice, its application may be arbitrary. Because an individual with normal liver parameters would be allocated to Child-Pugh class A, an inappropriately low starting dose, as recommended within the prescribing information for Child-Pugh class A, may then be administered. Furthermore, mechanistically distinct types of liver pathology may influence drug metabolism in diverse ways. For example, in PK studies with gefitinib, patients with moderate and severe hepatic impairment due to liver metastases had no clinically relevant differences in drug exposure; patients characterized as Child-Pugh class B and C secondary to cirrhosis, however, had a significant increase in gefitinib exposure [23]. In this analysis, we observed a numerically higher rate of grade 3/4 treatment-emergent adverse events requiring eribulin dose modification in patients with raised bilirubin levels in the presence of liver metastases (19/23, 86%) than in patients with raised bilirubin and no liver metastases (5/11, 45%).

Concerns have been raised regarding the use of Child-Pugh criteria to guide dosing in patients with metastatic cancer [24]. As such, an alternative system based on LFTs such as the National Cancer Institute–Organ Dysfunction Working Group (NCI-ODWG) [25] may be more relevant. The NCI-ODWG criteria are based on levels of bilirubin and AST. Increased bilirubin up to 1.5 times upper limit of normal or AST greater than the upper limit of normal would categorize a patient as having mild hepatic dysfunction and this correlates with Child-Pugh class A [25]. However, the equal weighting applied by this score to bilirubin and AST in defining mild hepatic dysfunction may not be appropriate for a drug such as eribulin that is eliminated by biliary excretion as unchanged drug. Our data suggest that a patient allocated to the NCI-ODWG mild category because they have elevated bilirubin levels has a substantial chance of toxicity and dose reduction. On the other hand, a patient allocated to the same group because of raised AST levels most likely does not.

Some limitations of this analysis are that it is retrospective, the subgroup of patients with elevated bilirubin was small (*n =* 34), and the studies did not collect relevant clinical and biochemical data to enable calculation of Child-Pugh score, precluding a comparison between bilirubin levels and Child-Pugh classes. Additionally, the efficacy data presented in this study should be interpreted with caution as results may have been impacted by factors that were not accounted for in the study, such as the volume of liver disease and previous lines of therapy.

Despite its limitations, this analysis suggests the need for improved guidance regarding eribulin dosing in patients with liver impairment. As such, we propose a dosing scheme based on the following: (1) the entry criteria for Studies 305 and 301, (2) dose modifications defined within the study protocols and the Summary of Product Characteristics, and (3) pooled LFT data from this analysis (Table 4). This guidance may be more aligned with clinical practice than guidance based on Child-Pugh criteria; however, it is unproven in a real-world setting. Moreover, this scheme was developed based on a relatively small sample size. As such, additional investigation will be necessary to validate this proposed scheme.

**Table 4:** Proposed Macpherson-Palmieri dosing scheme for eribulin mesylate

***Initial dosing***		
	**Clinical definition**	**Dosing recommendations**
Group 1	Adequate liver function^a^	1.4 mg/m^2^ (equivalent to 1.23 mg/m^2^ eribulin [expressed as free base])
Group 2	Bilirubin level: > ULN ≤ 1.5 × ULN	1.1 mg/m^2^
Group 3	Bilirubin level: > 1.5 × ULN or ALT/AST: ≥ 3 × ULN (for liver metastases, ALT/AST: ≥ 5 × ULN)	0.7 mg/m^2 b^
***Subsequent dosing: day 8***		
	**Clinical finding**	**Dosing modification**
Group 1	ANC < 1.0 × 10^9^/L and/or platelet count < 75 × 10^9^/L; or nonhematological AE > grade 2^c^	Postpone treatment until recovery
Group 2	ANC < 1.0 × 10^9^/L and/or platelet count < 75 × 10^9^/L; or nonhematological AE > grade 2^c^	Postpone treatment until recovery
Group 3	ANC < 1.0 × 10^9^/L and/or platelet count < 75 × 10^9^/L; or nonhematological AE > grade 2^c^	Postpone treatment; omit day 8
***Recovery on day 15 or earlier***		
	**Clinical action**	**Dosing modification**
Group 1	Resume treatment on the day that recovery is observed (considered new day 8 of cycle)	1.1 mg/m^2^
Group 2	Resume treatment on the day that recovery is observed (considered new day 8 of cycle)	0.7 mg/m^2^
***Recovery after day 15***		
	**Clinical action**	**Dosing modification**
Group 1	Omit day 8 and resume scheduled treatment on day 1 of next cycle	1.1 mg/m^2^
Group 2	Omit day 8 and resume scheduled treatment on day 1 of next cycle	0.7 mg/m^2^
**Special considerations:**		
• **Grade 4 neutropenia > 7 days**		
• **Grade 3/4 neutropenia with fever or infection requiring treatment with growth factors and/or antibiotics**		
• **Grade 4 thrombocytopenia**		
• **Grade 3 thrombocytopenia requiring platelet and/or blood transfusion**		
• **Nonhematologic grade 3–4 toxicities**		
	**Clinical action**	**Dosing modification**
Group 1	Resume treatment on day 1 of next cycle	1.1 mg/m^2^
Group 2	Resume treatment on day 1 of next cycle	0.7 mg/m^2^
Group 3		Consider stopping treatment with eribulin mesylate
**Dose escalation**^**d**^		
	**Clinical action**	**Dose modification**
Group 2 and 3	If, following cycle 1 day 1 and cycle 1 day 8, there are no toxicity issues (especially hematological toxicities determined by ANC and platelets), consider increasing dose at the start of the next cycle Refer to day 8 of this table to assess hematological and nonhematological toxicities at the next dose level; reduce dose if necessary	Next dose level

This dosing scheme is the work of investigators (IM and CP). These unproven recommendations require further study; dosing recommendations are based on eribulin mesylate unless otherwise specified

^a^Defined as bilirubin ≤ ULN and AP, ALT, and AST ≤ 3 × ULN (in the presence of liver metastases: ≤ 5 × ULN); unless there are bone metastases, in which case liver-specific AP must be separated from the total and used to assess the liver function instead of the total AP. If AP is > 3 × ULN (in absence of liver metastases) or > 5 × ULN (in presence of liver metastases) *and* patient also is known to have bone metastases, the liver-specific AP must be separated from the total and used to assess the liver function instead of the total AP

^b^There are no data for this group given that such patients were excluded from the study. Given the lack of clinical data, extreme caution should be applied to the use of eribulin within this group and clinical judgment should be used. If eribulin is used, then close monitoring for toxicities is required

^c^Except inadequately treated nausea and/or vomiting

^d^To be considered when treatment was initiated at a lower starting dose

*AE*, adverse event; *ALT*, alanine transaminase; *ANC*, absolute neutrophil count; *AP*, alkaline phosphatase; *AST*, aspartate transaminase; *ULN*, upper limit of normal

## Conclusions

This post hoc pooled analysis of LFT data from 1062 patients enrolled in Studies 301 and 305 provides evidence that elevated bilirubin levels at baseline are associated with a reduction in dose and actual dose intensity of eribulin, a higher proportion of grade 3 and 4 toxicities, and a numerically lower ORR. These findings highlight a need for caution regarding the dosing of eribulin in patients with bilirubin levels outside the normal range. Currently, the guidance for dosing eribulin in patients with hepatic impairment is based on the Child-Pugh criteria; however, there are limitations to using this approach in patients with cancer. Here, we propose an alternative dosing schema for eribulin which is based on information contained within the protocols for Studies 301 and 305 and the Summary of Product Characteristics, as well as the current pooled analysis of LFT data. While this dosing schema does require further validation, it is more reflective of the information used within pivotal metastatic breast cancer studies with regard to liver function and eribulin dosing.

## Supplementary Information

**Additional file 1: Supplementary Table S1.** Eribulin mesylate dose modifications and exposure in patients with or without liver lesion involvement (safety population).

**Additional file 2: Supplementary Table S2.** TEAEs occurring in >10% of patients in any group (safety population).

**Additional file 3: Supplementary Table S3.** TEAEs leading to dose modifications (reductions, delays, or interruptions) in patients with or without liver lesion involvement and by CTCAE grade (safety population).

**Additional file 4: Supplementary Table S4.** Neutrophil/granulocyte CTCAE grade at baseline and after cycle 1 in patients with liver impairment.

**Additional file 5: Supplementary Table S5.** Overall survival in patients with liver impairment (ITT population).

**Additional file 6: Supplementary Table S6.** Overall survival in patients with or without liver involvement (ITT population).

## Data Availability

The datasets generated during and/or analyzed during the current study are not publicly available because of commercial confidentiality but are available from the corresponding author on reasonable request.

## Abbreviations

ALT: Alanine aminotransferase
AP: Alkaline phosphatase
AST: Aspartate aminotransferase
AUC_0-∞_: Area under the curve from zero to infinity
CBR: Clinical benefit rate
CR: Complete response
CTCAE: Common Terminology Criteria for Adverse Events
ECOG: Eastern Cooperative Oncology Group
EMA: European Medicines Agency
FDA: Food and Drug Administration
HER2: Human epidermal growth factor receptor 2
ITT: Intent to treat
LFT: Liver-function test
MBC: Metastatic breast cancer
NCI-ODWG: National Cancer Institute–Organ Dysfunction Working Group
ORR: Objective response rate
OS: Overall survival
PK: Pharmacokinetics
PR: Partial response
TEAEs: Treatment-emergent adverse events
ULN: Upper limits of normal

## References

[1] Towle MJ, Salvato KA, Budrow J, Wels BF, Kuznetsov G, Aalfs KK (2001). In vitro and in vivo anticancer activities of synthetic macrocyclic ketone analogues of halichondrin B. Cancer Res.

[2] Jordan MA, Kamath K, Manna T, Okouneva T, Miller HP, Davis C (2005). The primary antimitotic mechanism of action of the synthetic halichondrin E7389 is suppression of microtubule growth. Mol Cancer Ther.

[3] Smith JA, Wilson L, Azarenko O, Zhu X, Lewis BM, Littlefield BA (2010). Eribulin binds at microtubule ends to a single site on tubulin to suppress dynamic instability. Biochemistry..

[4] Cortes J, O'Shaughnessy J, Loesch D, Blum JL, Vahdat LT, Petrakova K (2011). Eribulin monotherapy versus treatment of physician’s choice in patients with metastatic breast cancer (EMBRACE): a phase 3 open-label randomised study. Lancet..

[5] Kaufman PA, Awada A, Twelves C, Yelle L, Perez EA, Velikova G (2015). Phase III open-label randomized study of eribulin mesylate versus capecitabine in patients with locally advanced or metastatic breast cancer previously treated with an anthracycline and a taxane. J Clin Oncol.

[6] Halaven (eribulin mesylate) injection [prescribing information]. Woodcliff Lake: Eisai Inc.; 2016. https://www.accessdata.fda.gov/drugsatfda_docs/label/2016/201532s015lbl.pdf. Accessed 26 Feb 2021.

[7] Halaven 0.44 mg/ml solution for injection [summary of product characteristics]. Hertfordshire: Eisai Europe Limited. https://www.medicines.org.uk/emc/medicine/24382/SPC/Halaven+0.44+mg+ml+solution+for+injection/. Accessed 26 Feb 2021.

[8] Dubbelman AC, Rosing H, Jansen RS, Mergui-Roelvink M, Huitema AD, Koetz B (2012). Mass balance study of [^14^C] eribulin in patients with advanced solid tumors. Drug Metab Dispos.

[9] Zhang ZY, King BM, Pelletier RD, Wong YN (2008). Delineation of the interactions between the chemotherapeutic agent eribulin mesylate (E7389) and human CYP3A4. Cancer Chemother Pharmacol.

[10] Devriese LA, Witteveen PO, Marchetti S, Mergui-Roelvink M, Reyderman L, Wanders J (2012). Pharmacokinetics of eribulin mesylate in patients with solid tumors and hepatic impairment. Cancer Chemother Pharmacol.

[11] Child CG, Turcotte JG, Child CG (1964). Surgery and portal hypertension. Liver and portal hypertension.

[12] Pugh RN, Murray-Lyon IM, Dawson JL, Pietroni MC, Williams R (1973). Transection of the oesophagus for bleeding oesophageal varices. Br J Surg.

[13] European Medicines Agency, Committee for Medicinal Products for Human Use (CHMP). Guideline on the evaluation of the pharmacokinetics of medicinal products in patients with impaired hepatic function. 2005. http://www.ema.europa.eu/docs/en_GB/document_library/Scientific_guideline/2009/09/WC500003122.pdf. Accessed 31 July 2020.

[14] U.S. Department of Health and Human Services, Food and Drug Administration, Center for Drug Evaluation and Research (CDER), Center for Biologics Evaluation and Research (CBER). Guidance for Industry: Pharmacokinetics in patients with impaired hepatic function: study design, data analysis, and impact on dosing and labeling. 2003. http://www.fda.gov/downloads/Drugs/GuidanceComplianceRegulatoryInformation/Guidances/ucm072123.pdf. Accessed 20 July 2020.

[15] Krens SD, Lassche G, Jansman FGA, Desar IME, Lankheet NAG, Burger DM (2019). Dose recommendations for anticancer drugs in patients with renal or hepatic impairment. Lancet Oncol.

[16] Twelves C, Cortes J, Vahdat L, Olivo M, He Y, Kaufman PA (2014). Efficacy of eribulin in women with metastatic breast cancer: a pooled analysis of two phase 3 studies. Breast Cancer Res Treat.

[17] Devriese LA, Mergui-Roelvink M, Wanders J, Jenner A, Edwards G, Reyderman L (2013). Eribulin mesylate pharmacokinetics in patients with solid tumors receiving repeated oral ketoconazole. Investig New Drugs.

[18] Reyderman L, Gupta A, Pelletier RD, Wong N (2011). Eribulin and cytochrome P450 effectors: in vitro studies and population pharmacokinetic–pharmacodynamic analysis. EJC Suppl.

[19] Devriese LA, Witteveen PE, Wanders J, Law K, Edwards G, Reyderman L (2013). Pharmacokinetics of eribulin mesylate in patients with solid tumours receiving repeated oral rifampicin. Br J Clin Pharmacol.

[20] Majid O, Gupta A, Reyderman L, Olivo M, Hussein Z (2014). Population pharmacometric analyses of eribulin in patients with locally advanced or metastatic breast cancer previously treated with anthracyclines and taxanes. J Clin Pharmacol.

[21] Edwards BK, Noone AM, Mariotto AB, Simard EP, Boscoe FP, Henley SJ (2014). Annual report to the nation on the status of cancer, 1975-2010, featuring prevalence of comorbidity and impact on survival among persons with lung, colorectal, breast, or prostate cancer. Cancer..

[22] Lin Z, Yan S, Zhang J, Pan Q (2018). A nomogram for distinction and potential prediction of liver metastasis in breast cancer patients. J Cancer.

[23] Horak J, White J, Harris AL, Verrill M, Carmichael J, Holt A (2011). The effect of different etiologies of hepatic impairment on the pharmacokinetics of gefitinib. Cancer Chemother Pharmacol.

[24] Palmieri C, Macpherson I (2019). Use of the Child-Pugh score in anticancer drug dosing decision making: proceed with caution. Lancet Oncol..

[25] Patel H, Egorin MJ, Remick SC, Mulkerin D, Takimoto CH, Doroshow JH et al. Comparison of Child-Pugh (CP) criteria and NCI organ dysfunction working group (NCI-ODWG) criteria for hepatic dysfunction (HD): Implications for chemotherapy dosing [abstract]. J Clin Oncol. 2004;22(14 Suppl): Abstract 6051.

