# Supplementary material for: Eribulin, Child-Pugh score, and liver-function tests: lessons from pivotal breast cancer studies 301 and 305
Source: Breast Cancer Res. 2021 Mar 18;23:33. doi: 10.1186/s13058-021-01407-w (PMC7977154; doi:10.1186/s13058-021-01407-w)
Supplement: Supplementary file 1 — Additional file 1: Supplementary Table S1. Eribulin mesylate dose modifications and exposure in patients with or without liver lesion involvement (safety population). [file 13058_2021_1407_MOESM1_ESM.pdf]

SUPPLEMENTARY TABLES

Supplementary Table S1. Eribulin mesylate dose modifications and exposure in patients with or without liver lesion involvement (safety population)

Parameter	With Liver Involvement				Without Liver Involvement			
	Normal	Liver Impairment			Normal	Liver Impairment		
	A (n=204)	B (n=210)	C (n=294)	D (n=23)	A (n=336)	B (n=82)	C (n=146)	D (n=11)
**Duration of treatment**								
**n**	204	210	294	23	336	82	146	11
**Days, mean (SD)**	154.74 (125.42)	143.29 (102.81)	137.23 (99.45)	119.70 (62.81)	173.03 (182.53)	173.09 (161.23)	146.36 (137.20)	147.73 (98.73)
**Number of cycles completed in study**								
**Median (range)**	6.0 (1.0, 41.0)	6.0 (1.0, 27.0)	5.0 (1.0, 27.0)	5.0 (1.0, 11.0)	6.0 (1.0, 65.0)	6.5 (1.0, 38.0)	5.0 (1.0, 38.0)	4.0 (2.0, 15.0)
**Actual dose intensity**								
**n**	204	210	294	22	336	82	146	11
**Mean, $mg/m^2/week$**	0.83	0.78	0.78	0.64	0.81	0.77	0.77	0.67

**(SD)**	(0.134)	(0.155)	(0.158)	(0.165)	(0.139)	(0.164)	(0.162)	(0.217)
**Patients who received dose**								
**n (%)**	204 (100)	210 (100)	294 (100)	23 (100)	336 (100)	82 (100)	146 (100)	11 (100)
**Dose reduction**								
**n (%)**	48 (23.5)	74 (35.2)	106 (36.1)	15 (65.2)	93 (27.7)	27 (32.9)	47 (32.2)	6 (54.6)
**Dose delay**								
**n (%)**	83 (40.7)	105 (50.0)	147 (50.0)	17 (73.9)	140 (41.7)	36 (43.9)	65 (44.5)	6 (54.6)

Group A, no liver impairment; group B, increased AST and/or ALT only; group C, any abnormality except increased bilirubin level; group D, increased bilirubin level.

ALT, alanine aminotransferase; AST, aspartate aminotransferase; SD, standard deviation.

Supplementary Table S2. TEAEs occurring in >10% of patients in any group (safety population)

TEAE, n (%)	Normal		Liver Impairment					
	Group A (n=540)		Group B (n=292)		Group C (n=440)		Group D (n=34)	
	All	Grade ≥3	All	Grade ≥3	All	Grade ≥3	All	Grade ≥3
**Any TEAE**	520 (96.3)	328 (60.7)	278 (95.2)	209 (71.6)	424 (96.4)	326 (74.1)	34 (100)	29 (85.3)
**Neutropenia**	260 (48.2)	216 (40.0)	168 (57.5)	147 (50.3)	253 (57.5)	222 (50.5)	25 (73.5)	24 (70.6)
**Alopecia**	204 (37.8)	0	131 (44.9)	0	183 (41.6)	0	16 (47.1)	0
**Nausea**	140 (25.9)	2 (0.4)	96 (32.9)	1 (0.3)	143 (32.5)	5 (1.1)	9 (26.5)	0
**Leukopenia**	145 (26.9)	70 (13.0)	72 (24.7)	42 (14.4)	121 (27.5)	66 (15.0)	11 (32.4)	8 (23.5)
**Asthenia**	96 (17.8)	20 (3.7)	56 (19.2)	16 (5.5)	101 (23.0)	30 (6.8)	10 (29.4)	3 (8.8)
**Decreased appetite**	89 (16.5)	0	48 (16.4)	3 (1.0)	82 (18.6)	5 (1.1)	9 (26.5)	0
**Diarrhea**	84 (15.6)	5 (0.9)	49 (16.8)	1 (0.3)	75 (17.1)	1 (0.2)	9 (26.5)	0
**Fatigue**	122 (22.6)	10 (1.9)	75 (25.7)	11 (3.8)	106 (24.1)	18 (4.1)	8 (23.5)	0
**Pyrexia**	84 (15.6)	1 (0.2)	52 (17.8)	0	81 (18.4)	2 (0.5)	9 (26.5)	0
**Anemia**	90 (16.7)	3 (0.6)	53 (18.2)	11 (3.8)	96 (21.8)	16 (3.6)	7 (20.6)	2 (5.9)
**Constipation**	68 (12.6)	1 (0.2)	62 (21.2)	1 (0.3)	90 (20.5)	4 (0.9)	5 (14.7)	0
**Vomiting**	69 (12.8)	2 (0.4)	51 (17.5)	1 (0.3)	80 (18.2)	5 (1.1)	7 (20.6)	0
**Headache**	80 (14.8)	4 (0.7)	56 (19.2)	2 (0.7)	79 (18.0)	2 (0.5)	4 (11.8)	0

**Dyspnea**	65 (12.0)	17 (3.2)	37 (12.7)	10 (3.4)	63 (14.3)	19 (4.3)	6 (17.7)	1 (2.9)
**Hypokalemia**	21 (3.9)	5 (0.9)	12 (4.1)	4 (1.4)	28 (6.4)	9 (2.1)	6 (17.7)	3 (8.8)
**Weight, decreased**	41 (7.6)	2 (0.4)	45 (15.4)	0	77 (17.5)	0	6 (17.7)	1 (2.9)
**Peripheral sensory neuropathy**	84 (15.6)	16 (3.0)	35 (12.0)	9 (3.1)	46 (10.5)	12 (2.7)	5 (14.7)	1 (2.9)
**Hyperbilirubinemia**	3 (0.6)	0	5 (1.7)	1 (0.3)	9 (2.1)	2 (0.5)	5 (14.7)	2 (5.9)
**Lacrimation, increased**	17 (3.2)	0	26 (8.9)	1 (0.3)	30 (6.8)	1 (0.2)	5 (14.7)	0
**Stomatitis**	25 (4.6)	1 (0.2)	18 (6.2)	1 (0.3)	34 (7.7)	4 (0.9)	5 (14.7)	1 (2.9)
**Back pain**	75 (13.9)	7 (1.3)	39 (13.4)	4 (1.4)	51 (11.6)	6 (1.4)	4 (11.8)	0
**Bone pain**	47 (8.7)	3 (0.6)	39 (13.4)	10 (3.4)	55 (12.5)	15 (3.4)	2 (5.9)	0
**Arthralgia**	66 (12.2)	5 (0.9)	33 (11.3)	1 (0.3)	42 (9.6)	1 (0.2)	2 (5.9)	0
**Cough**	60 (11.1)	2 (0.4)	35 (12.0)	1 (0.3)	51 (11.6)	2 (0.5)	4 (11.8)	0
**Paresthesia**	37 (6.9)	10 (1.9)	27 (9.3)	2 (0.7)	38 (8.6)	3 (0.7)	4 (11.8)	0
**Dry mouth**	21 (3.9)	0	12 (4.1)	0	19 (4.3)	0	4 (11.8)	0
**Epistaxis**	6 (1.1)	0	4 (1.4)	0	10 (2.3)	0	4 (11.8)	0
**ALT, increased**	29 (5.4)	12 (2.2)	33 (11.3)	14 (4.8)	41 (9.3)	15 (3.4)	1 (2.9)	0
**Pain in extremity**	56 (10.4)	2 (0.4)	32 (11.0)	1 (0.3)	45 (10.2)	5 (1.1)	0	0

Group A, no liver impairment; group B, increased AST and/or ALT only; group C, any abnormality except increased bilirubin; group D, increased bilirubin.

ALT, alanine aminotransferase; AST, aspartate aminotransferase; TEAE, treatment-emergent adverse event.

Supplementary Table S3. TEAEs leading to dose modifications (reductions, delays, or interruptions) in patients with or without liver lesion involvement and by CTCAE grade (safety population)

	With Liver Involvement				Without Liver Involvement			
	Normal	Liver Impairment			Normal	Liver Impairment		
	A (n=204)	B (n=210)	C (n=294)	D (n=23)	A (n=336)	B (n=82)	C (n=146)	D (n=11)
**Overall, n (%)**								
**Total**	85 (41.7)	114 (54.3)	154 (52.4)	21 (91.3)	146 (43.5)	45 (54.9)	77 (52.7)	5 (45.5)
**Grade 1**	3 (1.5)	7 (3.3)	8 (2.7)	2 (8.7)	5 (1.5)	4 (4.9)	5 (3.4)	0
**Grade 2**	21 (10.3)	15 (7.1)	18 (6.1)	0	26 (7.7)	10 (12.2)	16 (11.0)	0
**Grade 3**	38 (18.6)	53 (25.2)	73 (24.8)	6 (26.1)	73 (21.7)	23 (28.1)	41 (28.1)	4 (36.4)
**Grade 4**	23 (11.3)	39 (18.6)	55 (18.7)	13 (56.5)	42 (12.5)	8 (9.8)	15 (10.3)	1 (9.1)

Group A, no liver impairment; group B, increased AST and/or ALT only; group C, any abnormality except increased bilirubin; group D, increased bilirubin.

ALT, alanine aminotransferase; AST, aspartate aminotransferase; CTCAE, Common Terminology Criteria for Adverse Events; TEAE, treatment-emergent adverse event.

Supplementary Table S4. Neutrophil/granulocyte CTCAE grade at baseline and after cycle 1 in patients with liver impairment

n (%)	Normal	Liver Impairment		
	Group A (n=540)	Group B (n=292)	Group C (n=440)	Group D (n=34)
**Baseline**				
**n**	538	286	433	34
**Any grade**	26 (4.8)	14 (4.9)	17 (3.9)	2 (5.9)
**Grade 1**	25 (4.6)	13 (4.5)	16 (3.7)	1 (2.9)
**Grade 2**	1 (0.2)	1 (0.3)	1 (0.2)	1 (2.9)
**Cycle 1 day 8**				
**n**	532	275	414	33
**Any grade**	154 (28.9)	106 (38.5)	155 (37.4)	10 (30.3)
**Grade 1**	68 (12.8)	35 (12.7)	49 (11.8)	2 (6.1)
**Grade 2**	64 (12.0)	43 (15.6)	65 (15.7)	4 (12.1)
**Grade 3**	16 (3.0)	17 (6.2)	27 (6.5)	1 (3.0)
**Grade 4**	6 (1.1)	11 (4.0)	14 (3.4)	3 (9.1)
**Cycle 2 day 8**				
**n**	507	266	393	28
**Any grade**	77 (15.2)	59 (22.2)	83 (21.1)	4 (14.3)
**Grade 1**	42 (8.3)	31 (11.7)	40 (10.2)	2 (7.1)
**Grade 2**	29 (5.7)	22 (8.3)	33 (8.4)	2 (7.1)
**Grade 3**	6 (1.2)	3 (1.1)	6 (1.5)	0
**Grade 4**	0	3 (1.1)	4 (1.0)	0

Cycle 3 day 8				
**n**	427	222	320	26
**Any grade**	66 (15.5)	42 (18.9)	56 (17.5)	4 (15.4)
**Grade 1**	38 (8.9)	21 (9.5)	27 (8.4)	3 (11.5)
**Grade 2**	20 (4.7)	16 (7.2)	21 (6.6)	1 (3.8)
**Grade 3**	7 (1.6)	4 (1.8)	6 (1.9)	0
**Grade 4**	1 (0.2)	1 (0.5)	2 (0.6)	0

Percentages based on the number of subjects with nonmissing data at each visit within each laboratory test.

Group A, no liver impairment; group B, increased AST and/or ALT only; group C, any abnormality except increased bilirubin; group D, increased bilirubin.

ALT, alanine aminotransferase; AST, aspartate aminotransferase; CTCAE, Common Terminology Criteria for Adverse Events.

Supplementary Table S5. Overall survival in patients with liver impairment (ITT population)

	Normal	Liver Impairment Subgroup		
	**Group A (n=546)**	**Group B (n=294)**	**Group C (n=443)**	**Group D (n=34)**
**Median OS, months (95% CI)**	17.5 (15.9–18.8)	13.2 (11.7–14.5)	12.3 (11.1–13.6)	12.3 (8.0–16.1)
**OS rate (95% CI)[a]**				
**1 year**	0.672 (0.633–0.712)	0.542 (0.485–0.599)	0.505 (0.458–0.552)	0.529 (0.362–0.739)
**2 years**	0.370 (0.328–0.412)	0.199 (0.151–0.248)	0.183 (0.145–0.221)	0.206 (0.070–0.342)
**3 years**	0.212 (0.170–0.253)	0.098 (0.055–0.141)	0.085 (0.051–0.118)	0 (NE–NE)

[a]OS rate at 1, 2, and 3 years and the 95% CIs were calculated using Kaplan–Meier estimate and Greenwood's formula.

Group A, no liver impairment; group B, increased AST and/or ALT only; group C, any abnormality except increased bilirubin; group D, increased bilirubin.

ALT, alanine aminotransferase; AST, aspartate aminotransferase; CI, confidence interval; ITT, intent to treat; NE, nonestimable; OS, overall survival.

Supplementary Table S6. Overall survival in patients with or without liver involvement (ITT population)

	With Liver Involvement				Without Liver Involvement			
	Normal	Liver Impairment			Normal	Liver Impairment		
	Group A (n=207)	Group B (n=211)	Group C (n=296)	Group D (n=23)	Group A (n=339)	Group B (n=83)	Group C (n=147)	Group D (n=11)
**Overall, n (%)**								
**Median OS, months (95% CI)**	16.0 (14.6–18.0)	11.9 (10.0–13.2)	11.6 (10.0–13.1)	9.5 (6.9–16.3)	18.6 (16.5–22.2)	18.1 (14.9–21.2)	14.5 (11.2–16.7)	12.6 (6.3–16.3)
**OS rate (95% CI)[a]**								
**1 year**	0.640 (0.574–0.706)	0.487 (0.419–0.554)	0.475 (0.418–0.532)	0.435 (0.232–0.637)	0.692 (0.643–0.742)	0.683 (0.582–0.784)	0.565 (0.484–0.686)	0.727 (0.464–0.990)
**2 years**	0.305 (0.238–0.371)	0.144 (0.094–0.195)	0.149 (0.105–0.192)	0.217 (0.049–0.386)	0.408 (0.354–0.461)	0.340 (0.233–0.448)	0.251 (0.178–0.324)	0.182 (0.000–0.410)
**3 years**	0.146 (0.084–0.208)	0.081 (0.039–0.123)	0.081 (0.044–0.119)	0 (NE–NE)	0.248 (0.193–0.303)	0.137 (0.029–0.244)	0.097 (0.034–0.161)	0 (NE–NE)

[a]OS rates at 1, 2, and 3 years and the 95% CIs were calculated using Kaplan–Meier estimate and Greenwood's formula.

Group A, no liver impairment; group B, increased AST and/or ALT only; group C, any abnormality except increased bilirubin; group D, increased bilirubin.

ALT, alanine aminotransferase; AST, aspartate aminotransferase; CI, confidence interval; ITT, intent to treat; NE, nonestimable; OS, overall survival.
