# Supplementary material for: Eribulin, Child-Pugh score, and liver-function tests: lessons from pivotal breast cancer studies 301 and 305
Source: Breast Cancer Res. 2021 Mar 18;23:33. doi: 10.1186/s13058-021-01407-w (PMC7977154; doi:10.1186/s13058-021-01407-w)
Supplement: Supplementary file 2 — Additional file 2: Supplementary Table S2. TEAEs occurring in >10% of patients in any group (safety population). [file 13058_2021_1407_MOESM2_ESM.pdf]

Supplementary Table S2. TEAEs occurring in >10% of patients in any group (safety population)

TEAE, n (%)	Normal		Liver Impairment					
	Group A (n=540)		Group B (n=292)		Group C (n=440)		Group D (n=34)	
	All	Grade ≥3	All	Grade ≥3	All	Grade ≥3	All	Grade ≥3
**Any TEAE**	520 (96.3)	328 (60.7)	278 (95.2)	209 (71.6)	424 (96.4)	326 (74.1)	34 (100)	29 (85.3)
**Neutropenia**	260 (48.2)	216 (40.0)	168 (57.5)	147 (50.3)	253 (57.5)	222 (50.5)	25 (73.5)	24 (70.6)
**Alopecia**	204 (37.8)	0	131 (44.9)	0	183 (41.6)	0	16 (47.1)	0
**Nausea**	140 (25.9)	2 (0.4)	96 (32.9)	1 (0.3)	143 (32.5)	5 (1.1)	9 (26.5)	0
**Leukopenia**	145 (26.9)	70 (13.0)	72 (24.7)	42 (14.4)	121 (27.5)	66 (15.0)	11 (32.4)	8 (23.5)
**Asthenia**	96 (17.8)	20 (3.7)	56 (19.2)	16 (5.5)	101 (23.0)	30 (6.8)	10 (29.4)	3 (8.8)
**Decreased appetite**	89 (16.5)	0	48 (16.4)	3 (1.0)	82 (18.6)	5 (1.1)	9 (26.5)	0
**Diarrhea**	84 (15.6)	5 (0.9)	49 (16.8)	1 (0.3)	75 (17.1)	1 (0.2)	9 (26.5)	0
**Fatigue**	122 (22.6)	10 (1.9)	75 (25.7)	11 (3.8)	106 (24.1)	18 (4.1)	8 (23.5)	0
**Pyrexia**	84 (15.6)	1 (0.2)	52 (17.8)	0	81 (18.4)	2 (0.5)	9 (26.5)	0
**Anemia**	90 (16.7)	3 (0.6)	53 (18.2)	11 (3.8)	96 (21.8)	16 (3.6)	7 (20.6)	2 (5.9)
**Constipation**	68 (12.6)	1 (0.2)	62 (21.2)	1 (0.3)	90 (20.5)	4 (0.9)	5 (14.7)	0
**Vomiting**	69 (12.8)	2 (0.4)	51 (17.5)	1 (0.3)	80 (18.2)	5 (1.1)	7 (20.6)	0

**Headache**	80 (14.8)	4 (0.7)	56 (19.2)	2 (0.7)	79 (18.0)	2 (0.5)	4 (11.8)	0
**Dyspnea**	65 (12.0)	17 (3.2)	37 (12.7)	10 (3.4)	63 (14.3)	19 (4.3)	6 (17.7)	1 (2.9)
**Hypokalemia**	21 (3.9)	5 (0.9)	12 (4.1)	4 (1.4)	28 (6.4)	9 (2.1)	6 (17.7)	3 (8.8)
**Weight, decreased**	41 (7.6)	2 (0.4)	45 (15.4)	0	77 (17.5)	0	6 (17.7)	1 (2.9)
**Peripheral sensory neuropathy**	84 (15.6)	16 (3.0)	35 (12.0)	9 (3.1)	46 (10.5)	12 (2.7)	5 (14.7)	1 (2.9)
**Hyperbilirubinemia**	3 (0.6)	0	5 (1.7)	1 (0.3)	9 (2.1)	2 (0.5)	5 (14.7)	2 (5.9)
**Lacrimation, increased**	17 (3.2)	0	26 (8.9)	1 (0.3)	30 (6.8)	1 (0.2)	5 (14.7)	0
**Stomatitis**	25 (4.6)	1 (0.2)	18 (6.2)	1 (0.3)	34 (7.7)	4 (0.9)	5 (14.7)	1 (2.9)
**Back pain**	75 (13.9)	7 (1.3)	39 (13.4)	4 (1.4)	51 (11.6)	6 (1.4)	4 (11.8)	0
**Bone pain**	47 (8.7)	3 (0.6)	39 (13.4)	10 (3.4)	55 (12.5)	15 (3.4)	2 (5.9)	0
**Arthralgia**	66 (12.2)	5 (0.9)	33 (11.3)	1 (0.3)	42 (9.6)	1 (0.2)	2 (5.9)	0
**Cough**	60 (11.1)	2 (0.4)	35 (12.0)	1 (0.3)	51 (11.6)	2 (0.5)	4 (11.8)	0
**Paraesthesia**	37 (6.9)	10 (1.9)	27 (9.3)	2 (0.7)	38 (8.6)	3 (0.7)	4 (11.8)	0
**Dry mouth**	21 (3.9)	0	12 (4.1)	0	19 (4.3)	0	4 (11.8)	0
**Epistaxis**	6 (1.1)	0	4 (1.4)	0	10 (2.3)	0	4 (11.8)	0
**ALT, increased**	29 (5.4)	12 (2.2)	33 (11.3)	14 (4.8)	41 (9.3)	15 (3.4)	1 (2.9)	0
**Pain in extremity**	56 (10.4)	2 (0.4)	32 (11.0)	1 (0.3)	45 (10.2)	5 (1.1)	0	0

Group A, no liver impairment; group B, increased AST and/or ALT only; group C, any abnormality except increased bilirubin; group D, increased bilirubin.

ALT, alanine aminotransferase; AST, aspartate aminotransferase; TEAE, treatment-emergent adverse event.
