# Supplementary material for: Eribulin, Child-Pugh score, and liver-function tests: lessons from pivotal breast cancer studies 301 and 305
Source: Breast Cancer Res. 2021 Mar 18;23:33. doi: 10.1186/s13058-021-01407-w (PMC7977154; doi:10.1186/s13058-021-01407-w)
Supplement: Supplementary file 3 — Additional file 3: Supplementary Table S3. TEAEs leading to dose modifications (reductions, delays, or interruptions) in patients with or without liver lesion involvement and by CTCAE grade (safety population). [file 13058_2021_1407_MOESM3_ESM.pdf]

Supplementary Table S3. TEAEs leading to dose modifications (reductions, delays, or interruptions) in patients with or without liver lesion involvement and by CTCAE grade (safety population)

	With Liver Involvement				Without Liver Involvement			
	Normal	Liver Impairment			Normal	Liver Impairment		
	A (n=204)	B (n=210)	C (n=294)	D (n=23)	A (n=336)	B (n=82)	C (n=146)	D (n=11)
**Overall, n (%)**								
**Total**	85 (41.7)	114 (54.3)	154 (52.4)	21 (91.3)	146 (43.5)	45 (54.9)	77 (52.7)	5 (45.5)
**Grade 1**	3 (1.5)	7 (3.3)	8 (2.7)	2 (8.7)	5 (1.5)	4 (4.9)	5 (3.4)	0
**Grade 2**	21 (10.3)	15 (7.1)	18 (6.1)	0	26 (7.7)	10 (12.2)	16 (11.0)	0
**Grade 3**	38 (18.6)	53 (25.2)	73 (24.8)	6 (26.1)	73 (21.7)	23 (28.1)	41 (28.1)	4 (36.4)
**Grade 4**	23 (11.3)	39 (18.6)	55 (18.7)	13 (56.5)	42 (12.5)	8 (9.8)	15 (10.3)	1 (9.1)

Group A, no liver impairment; group B, increased AST and/or ALT only; group C, any abnormality except increased bilirubin; group D, increased bilirubin.

CTCAE, Common Terminology Criteria for Adverse Events; TEAE, treatment-emergent adverse event.
