# Supplementary material for: Eribulin, Child-Pugh score, and liver-function tests: lessons from pivotal breast cancer studies 301 and 305
Source: Breast Cancer Res. 2021 Mar 18;23:33. doi: 10.1186/s13058-021-01407-w (PMC7977154; doi:10.1186/s13058-021-01407-w)
Supplement: Supplementary file 4 — Additional file 4: Supplementary Table S4. Neutrophil/granulocyte CTCAE grade at baseline and after cycle 1 in patients with liver impairment. [file 13058_2021_1407_MOESM4_ESM.pdf]

Supplementary Table S4. Neutrophil/granulocyte CTCAE grade at baseline and after cycle 1 in patients with liver impairment

n (%)	Normal<br>Group A<br>(n=540)	Liver Impairment<br>Group B<br>(n=292)	Group C<br>(n=440)	Group D<br>(n=34)
**Baseline**				
**n**	538	286	433	34
**Any grade**	26 (4.8)	14 (4.9)	17 (3.9)	2 (5.9)
**Grade 1**	25 (4.6)	13 (4.5)	16 (3.7)	1 (2.9)
**Grade 2**	1 (0.2)	1 (0.3)	1 (0.2)	1 (2.9)
**Cycle 1 day 8**				
**n**	532	275	414	33
**Any grade**	154 (28.9)	106 (38.5)	155 (37.4)	10 (30.3)
**Grade 1**	68 (12.8)	35 (12.7)	49 (11.8)	2 (6.1)
**Grade 2**	64 (12.0)	43 (15.6)	65 (15.7)	4 (12.1)
**Grade 3**	16 (3.0)	17 (6.2)	27 (6.5)	1 (3.0)
**Grade 4**	6 (1.1)	11 (4.0)	14 (3.4)	3 (9.1)
**Cycle 2 day 8**				
**n**	507	266	393	28
**Any grade**	77 (15.2)	59 (22.2)	83 (21.1)	4 (14.3)
**Grade 1**	42 (8.3)	31 (11.7)	40 (10.2)	2 (7.1)
**Grade 2**	29 (5.7)	22 (8.3)	33 (8.4)	2 (7.1)
**Grade 3**	6 (1.2)	3 (1.1)	6 (1.5)	0
**Grade 4**	0	3 (1.1)	4 (1.0)	0
**Cycle 3 day 8**				
**n**	427	222	320	26
**Any grade**	66 (15.5)	42 (18.9)	56 (17.5)	4 (15.4)
**Grade 1**	38 (8.9)	21 (9.5)	27 (8.4)	3 (11.5)
**Grade 2**	20 (4.7)	16 (7.2)	21 (6.6)	1 (3.8)
**Grade 3**	7 (1.6)	4 (1.8)	6 (1.9)	0
**Grade 4**	1 (0.2)	1 (0.5)	2 (0.6)	0

Percentages based on the number of subjects with nonmissing data at each visit within each

laboratory test.

Group A, no liver impairment; group B, increased AST and/or ALT only; group C, any abnormality except increased bilirubin; group D, increased bilirubin.

ALT, alanine aminotransferase; AST, aspartate aminotransferase; CTCAE, Common Terminology Criteria for Adverse Events.
