# Supplementary material for: Eribulin, Child-Pugh score, and liver-function tests: lessons from pivotal breast cancer studies 301 and 305
Source: Breast Cancer Res. 2021 Mar 18;23:33. doi: 10.1186/s13058-021-01407-w (PMC7977154; doi:10.1186/s13058-021-01407-w)
Supplement: Supplementary file 5 — Additional file 5: Supplementary Table S5. Overall survival in patients with liver impairment (ITT population). [file 13058_2021_1407_MOESM5_ESM.pdf]

Supplementary Table S5. Overall survival in patients with liver impairment (ITT population)

	Normal	Liver Impairment Subgroup		
	Group A (n=546)	Group B (n=294)	Group C (n=443)	Group D (n=34)
**Median OS, months (95% CI)**	17.5 (15.9–18.8)	13.2 (11.7–14.5)	12.3 (11.1–13.6)	12.3 (8.0–16.1)
**OS rate (95% CI)[a]**				
**1 year**	0.672 (0.633–0.712)	0.542 (0.485–0.599)	0.505 (0.458–0.552)	0.529 (0.362–0.739)
**2 years**	0.370 (0.328–0.412)	0.199 (0.151–0.248)	0.183 (0.145–0.221)	0.206 (0.070–0.342)
**3 years**	0.212 (0.170–0.253)	0.098 (0.055–0.141)	0.085 (0.051–0.118)	0 (NE–NE)

[a]OS rate at 1, 2, and 3 years and the 95% CIs were calculated using Kaplan–Meier estimate and Greenwood's formula.

Group A, no liver impairment; group B, increased AST and/or ALT only; group C, any abnormality except increased bilirubin; group D, increased bilirubin.

ALT, alanine aminotransferase; AST, aspartate aminotransferase; CI, confidence interval; ITT, intent to treat; NE, nonestimable; OS, overall survival.
