# Supplementary material for: Eribulin, Child-Pugh score, and liver-function tests: lessons from pivotal breast cancer studies 301 and 305
Source: Breast Cancer Res. 2021 Mar 18;23:33. doi: 10.1186/s13058-021-01407-w (PMC7977154; doi:10.1186/s13058-021-01407-w)
Supplement: Supplementary file 6 — Additional file 6: Supplementary Table S6. Overall survival in patients with or without liver involvement (ITT population). [file 13058_2021_1407_MOESM6_ESM.pdf]

Supplementary Table S6. Overall survival in patients with or without liver involvement (ITT population)

	With Liver Involvement				Without Liver Involvement			
	Normal	Liver Impairment			Normal	Liver Impairment		
	Group A (n=207)	Group B (n=211)	Group C (n=296)	Group D (n=23)	Group A (n=339)	Group B (n=83)	Group C (n=147)	Group D (n=11)
**Overall, n (%)**								
**Median OS, months (95% CI)**	16.0 (14.6–18.0)	11.9 (10.0–13.2)	11.6 (10.0–13.1)	9.5 (6.9–16.3)	18.6 (16.5–22.2)	18.1 (14.9–21.2)	14.5 (11.2–16.7)	12.6 (6.3–16.3)
**OS rate (95% CI)[a]**								
**1 year**	0.640 (0.574–0.706)	0.487 (0.419–0.554)	0.475 (0.418–0.532)	0.435 (0.232–0.637)	0.692 (0.643–0.742)	0.683 (0.582–0.784)	0.565 (0.484–0.686)	0.727 (0.464–0.990)
**2 years**	0.305 (0.238–0.371)	0.144 (0.094–0.195)	0.149 (0.105–0.192)	0.217 (0.049–0.386)	0.408 (0.354–0.461)	0.340 (0.233–0.448)	0.251 (0.178–0.324)	0.182 (0.000–0.410)
**3 years**	0.146 (0.084–0.208)	0.081 (0.039–0.123)	0.081 (0.044–0.119)	0 (NE–NE)	0.248 (0.193–0.303)	0.137 (0.029–0.244)	0.097 (0.034–0.161)	0 (NE–NE)

[a]OS rates at 1, 2, and 3 years and the 95% CIs were calculated using Kaplan–Meier estimate and Greenwood's formula.

Group A, no liver impairment; group B, increased AST and/or ALT only; group C, any abnormality except increased bilirubin; group D, increased bilirubin.

ALT, alanine aminotransferase; AST, aspartate aminotransferase; CI, confidence interval; ITT, intent to treat; NE, nonestimable; OS, overall survival.
